# Measurements of knee rotation-reliability of an external device in vivo

**DOI:** 10.1186/1471-2474-12-291

**Published:** 2011-12-30

**Authors:** Per O Almquist, Charlotte Ekdahl, Per-Erik Isberg, Thomas Fridén

**Affiliations:** 1 Department of Health Sciences, Division of Physiotherapy, Lund University, Box 157, SE-221 00 Lund, Sweden; 2 Department of Statistics, Lund University, Lund, Sweden; 3 Faculty of Medicine, Lund University, Lund, Sweden

## Abstract

**Background:**

Knee rotation plays an important part in knee kinematics during weight-bearing activities. An external device for measuring knee rotation (the Rottometer) has previously been evaluated for validity by simultaneous measurements of skeletal movements with Roentgen Stereometric Analysis (RSA). The aim of this study was to investigate the reliability of the device.

**Method:**

The within-day and test-retest reliability as well as intertester reliability of the device in vivo was calculated. Torques of 3, 6 and 9 Nm and the examiner's apprehension of end-feel were used at 90°, 60° and 30° of knee flexion. Intraclass Correlation Coefficient _2,1 _(ICC _2,1_), 95% confidence interval (CI) of ICC and 95% CI between test trials and examiners were used as statistical tests.

**Result:**

ICC_2,1 _ranged from 0.50 to 0.94 at all three flexion angles at 6 and 9 Nm as well as end-feel, and from 0.22 to 0.75 at 3 Nm applied torque.

**Conclusion:**

The Rottometer was a reliable measurement instrument concerning knee rotation at the three different flexion angles (90°, 60° and 30°) with 6 and 9 Nm applied torques as well as the examiner's apprehension of end-feel. Three Nm was not a reliable torque. The most reliable measurements were made at 9 Nm applied torque.

## Background

Knee rotation play an important part in weight-bearing activities in the lower extremities, such as changing direction while walking, running and jumping [1-3]. It is possible that abnormal rotational kinematics may contribute to degenerative changes after ACL injuries [4], and abnormal femurotibial axial rotation has been described in patients with recurrent patellar dislocation using a radiographic technique [5]. However, the normal range of rotation of the knee in various healthy populations in vivo is not known, age and gender differences are not established, and knee rotation is hardly ever estimated in clinical practice. A non-invasive manual device to measure knee rotation evaluated regarding its validity and reliability could be of value as a complement to existing clinical tools and examination equipment. The device would aid in diagnosing rotational instability and provide an objective clinical assessment of normal knee kinematics and possible pathological findings after different knee injuries and disorders [6]. A few in vivo studies concerning knee rotation measuring devices, their validity and reliability, as well as knee rotation reference values have been reported [7-12]. Zarins et al. [11] measured knee rotation in a side-lying position. The knee was positioned and measured at 90°, 60°, 30°, 15° and 5° of flexion. The torque was applied manually by the examiner's estimation of end-feel but no grading of torques was reported. The test-retest reliability was calculated by two repeated measurements on each of thirteen subjects. High correlations for the total internal-external rotation (r = 0.93-0.96) were described at 90°, 60° and 30° of flexion. Shoemaker and Markolf [12] measured knee rotation in vivo combined with measurements of maximum isometrically generated tibial torque. Recordings were made in a sitting position and knee rotation was measured at 20° and 90° of flexion and a torque of 10 Nm was chosen. At 90°, seven tests were repeated on one subject, indicating good reproducibility (range 76°-83°). In a recent in vivo study [10] an external device was evaluated regarding inter-and test-retest reliability in 11 male subjects. The subjects were measured lying supine on an examination table, and were measured at 90° and 30° of knee flexion angle with 2, 4 and 6 Nm applied torques, and the total internal-external rotation was registered. The ICCs (Intraclass Correlation Coefficient) were all greater than 0.75 and the SEMs were all less than 2° in this study. Maudi et al. [8] used a knee rotatory kinaesthetic device to determine proprioceptive acuity for internal and external active rotation, and to measure active and passive rotation range of motion in vivo. To determine intra-and inter-observer reliability for active and passive rotation, 20 male subjects were recruited. Both intra-and inter-observer reliability were reported as good to excellent (ICC _1,2 _0.69-0.95). Measurements were made at 90° of flexion angle with 6 Nm applied torque. However, different flexion angles and different applied torques have been used and none of these measurement devices has been evaluated for validity by simultaneous measurements of actual skeletal movements in the same subjects. There is no consensus as to the most appropriate torque to use in knee rotation in vivo studies. In a previous study [13] a clinical device to measure the knee rotation in vivo (the Rottometer) was presented and evaluated for validity by simultaneous registrations with Roentgen Stereometric Analysis (RSA) [14]. The correlation between the two instruments was high (r^2 ^= 0.87-0.94) concerning total internal-external rotation. However, when evaluating knee rotation assessment tools, reliability must also be considered before they are used in research and clinical practice. The aim of this investigation was to evaluate the one-week-apart-, within-day-and intertester reliability of the Rottometer concerning total internal-external knee rotation, and to establish the most reliable torques and flexion angles.

## Methods

### The Rottometer (Figure 1) and testing procedure

**Figure 1:**
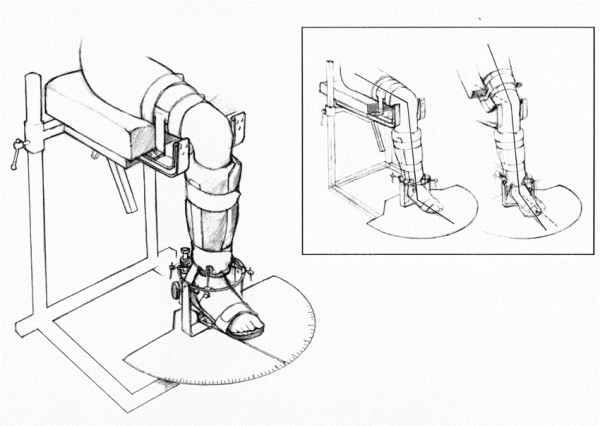
**The Rottometer with a test person fixed to the device ready to be measured**. In the frame: changes in flexion angles from 90° to 30° of knee flexion angle.

The Rottometer and details of the examination procedure and the fixation of the test persons to the device have been presented previously [13]. Measurements were made at 90°, 60° and 30° of knee flexion in each subject's both legs. Ninety degrees was chosen since the largest range of rotation has been reported at this knee flexion angle [1,2,15]. Sixty and 30° of flexion were chosen to study the rotation at more physiological flexion angles for weight-bearing activities. At each flexion angle, passive total internal-external rotation of the tibia in relation to the femur was measured. The neutral position of "zero degree" rotation was defined as each individual subject's resting position of the knee at each flexion angle. Total internal-external rotation was then measured from that zero position. The subjects were instructed to relax their muscles to allow the examiner to passively rotate the leg. An adjustable spanner was used to apply the different torques (Nm). It permitted measurements with various forces (Nm), and was calibrated and tested to provide torques reproducible within ± 3% (Rausol, Germany). The mean of three repeated measurements of internal and external rotation with 3, 6 and 9 Nm torque as well as the examiner's apprehension of end-feel [16] were recorded at each torque at each flexion angle (90°, 60° and 30°) in both knees. These forces were based on the results and experiences of several pilot studies before the main study. The examiner was instructed to stop the measuring procedure if the subject complained about major pain or discomfort at any applied torque at any angle.

### Reliability

#### On-week-apart intratester reliability

To evaluate the test-retest reliability of the knee joint rotation, one examiner (physiotherapist) measured 10 healthy subjects. The subjects were measured twice with a week's interval and these measurements were made at the same time of the day.

#### Within-day intratester reliability

To evaluate the within-day reliability, one examiner (physiotherapist) measured knee joint rotation in 10 healthy subjects. The subjects were measured twice, once in the morning and once in the afternoon, on the same day.

#### Intertester reliability

Ten healthy subjects participated in the study in order to evaluate the intertester reliability. They were measured twice on the same test occasion in random order by two independent examiners (physiotherapists). The first examiner fixed the subject to the Rottometer and carried out the measurements. After ten minutes' break after the first examination, the second examiner repeated the whole procedure on the same test person.

The examiners in the present study were physiotherapists and trained in using the measurement device before the study begun.

### Subjects

In the within-day and one-week-apart evaluation 10 subjects, six females (age range: 28-69 years) and four males (age range: 37-60 years) were measured. The measurements of intertester reliability were also performed on ten subjects, six females (range: 31-46 years) and four males (range: 24-35 years). These subjects had never undergone any knee, hip or foot surgery and had no documentation or history of prior major knee injuries (ligaments, fractures, meniscus). Each knee was considered as one unit and thus, twenty observations were used in the calculations. This study has been directed by the Helsinki Declaration and was approved by the Ethics Committee of Lund University

### Statistics

All statistical analyses of the data were performed using SPSS 14.0 statistics software. Statistical evaluation of the one-week-apart intratester-, within-day intratester and intertester reliability tests were made with Intraclass Correlation Coefficient_2,1 _(ICC_2,1_) [17], 95% Confidence Interval (CI) of the ICC [18]) and 95% CI between test trails and examiners.

## Results

### One-week-apart intratester reliability (Table 1)

**Table 1:** The results of the one-week-apart reliability evaluation of the external measurement device presented in means and Standard Deviations given in degrees (M 1 = measurement occasion one and M 2 = measurement occasion 2), and calculated with Intraclass Correlation Coefficient_2,1 _(ICC_2,1_), 95% Confidence Interval (CI) of ICC and 95% CI between M 1 and M 2.

Angle/applied torque	M 1 m ^± ^SD	M 2 m ^± ^SD	ICC_2,1_	95% CI of ICC	95% CI of M 1 and M 2
90°/3 Nm	30 ± 6	29 ± 6	0.49	0.04-0.77	-2.0-3.1

90°/6 Nm	56 ± 9	56 ± 5	0.67	0.31-0.86	-1.6-3.0

90°/9 Nm	76 ± 9	76 ± 7	0.80	0.56-0.92	-1.2-1.6

90°/End-feel	72 ± 8	71 ± 7	0.65	0.32-0.84	-1.3-2.2

60°/3 Nm	33 ± 8	33 ± 4	0.24	-0.15-0.59	-3.8-3.7

60°/6 Nm	58 ± 8	61 ± 9	0.50	0.11-0.76	-2.6-3.8

60°/9 Nm	77 ± 10	75 ± 8	0.82	0.60-0.92	-0.2-3.9

60°/End-feel	74 ± 8	73 ± 8	0.76	0.48-0.90	-0.4-3.2

30°/3 Nm	28 ± 8	29 ± 7	0.22	-0.48-0.40	-4.9-2.9

30°6 Nm	55 ± 10	53 ± 8	0.50	0.11-0.77	-0.4-3.5

30°/9 Nm	79 ± 12	77 ± 11	0.84	0.65-0.93	-0.6-4.6

30°/End-feel	78 ± 11	76 ± 9	0.82	0.64-0.93	-0.4-5.6

Calculations were made at three different flexion angles (90°, 60° and 30°) with four different applied torques (3, 6 and 9 Nm as well as the examiner's apprehension of end-feel) of the total internal-external knee joint rotation

The highest ICC_2,1 _(0.84) was registered at 30° of knee flexion angle with 9 Nm applied torque, and the lowest (0.22 and 0.24) at 60° and 30° with 3 Nm. The 95% CI between the two different tests was-4.9-5.6 at all four applied torques (3, 6 and 9 Nm and end-feel) at all three flexion angles.

### Within-day intratester reliability (Table 2)

**Table 2:** The results of the within-day reliability evaluation of the external measurement device presented in means and Standard Deviations given in degrees (M 1 = measurement occasion one and M 2 = measurement occasion 2), and calculated with Intraclass Correlation Coefficient_2,1 _(ICC_2,1_), 95% Confidence Interval (CI) of ICC and 95% CI between M 1 and M 2.

Angle/applied torque	M 1 m ^± ^SD	M 2 m ^± ^SD	ICC_2,1_	95% CI of ICC	95% CI of M 1 and M 2
90°/3 Nm	30 ± 6	33 ± 6	0.64	0.13-0.86	-5.1- -1.5

90°/6 Nm	56 ± 8	57 ± 7	0.73	0.43-0.87	-2.7-1.5

90°/9 Nm	76 ± 10	74 ± 10	0.79	0.54-0.91	-2.7-1.5

90°/End-feel	72 ± 8	73 ± 8	0.66	0.33-0.85	-3.4-0.9

60°/3 Nm	33 ± 8	37 ± 6	0.67	0.33-0.85	-0.6- -2.7

60°/6 Nm	58 ± 8	61 ± 9	0.76	0.50-0.90	0.4-4.4

60°/9 Nm	77 ± 10	78 ± 11	0.86	0.62-0.95	-2.6-1.3

60°/End-feel	74 ± 8	74 ± 9	0.90	0.75-0.96	-1.9-2.0

30°/3 Nm	24 ± 8	31 ± 9	0.59	0.21-0.81	-4.7- -0.2

30°/6 Nm	55 ± 10	56 ± 10	0.87	0.72-0.95	-2.7-1.2

30°/9 Nm	79 ± 12	77 ± 12	0.94	0.87-0.98	-0.4-9.9

30°/End-feel	78 ± 11	75 ± 11	0.83	0.61-0.93	-0.3-5.2

Calculations were made at three different flexion angles (90°, 60° and 30°) with four different applied torques (3, 6 and 9 Nm as well as the examiner's apprehension of end-feel) of the total internal-external knee joint rotation

The highest ICC_2,1 _was calculated as 0.94 at 9 Nm applied torque at 30° flexion angle, and the lowest (0.59) also at 30° but at 3 Nm. The 95% CI between the two different tests was-3.4-9.9 at 6 and 9 Nm as well as end-feel, and -6.0- -2.7 at 3 Nm at all three flexion angles.

### Intertester reliability (Table 3)

**Table 3:** The results of the inter-tester reliability evaluation of the external measurement device presented in means and Standard Deviations given in degrees (M 1 = measurement occasion one and M 2 = measurement occasion 2), and calculated with Intraclass Correlation Coefficient_2,1 _(ICC_2,1_), 95% Confidence Interval (CI) of ICC and 95% CI between M 1 and M 2.

Angle/applied torque	M 1 m ^± ^SD	M 2 m ^± ^SD	ICC_2,1_	95% CI of ICC	95% CI of M 1 and M 2
90°/3 Nm	30 ± 6	33 ± 7	0.49	0.09-0.76	-6.4-0.2

90°/6 Nm	53 ± 7	54 ± 8	0.87	0.71-0.95	-2.8-0.3

90°/9 Nm	70 ± 10	72 ± 10	0.85	0.68-0.94	-4.6-0.6

90°/End-feel	67 ± 8	66 ± 9	0.74	0.44-0.89	-1.7-3.8

60°/3 Nm	37 ± 7	38 ± 6	0.75	0.47-0.89	-3.5-0.9

60°/6 Nm	56 ± 8	58 ± 9	0.83	0.62-0.93	-3.6-0.4

60°/9 Nm	72 ± 10	74 ± 10	0.84	0.63-0.93	-3.7-0.1

60°/End-feel	71 ± 10	71 ± 9	0.77	0.51-0.90	-3.7-3.1

30°/3 Nm	28 ± 7	33 ± 8	0.52	0.10-0.78	-7.9- -2.1

30°/6 Nm	52 ± 10	55 ± 10	0.61	0.25-0.82	-6.8-0.2

30°/9 Nm	72 ± 11	74 ± 12	0.69	0.34-0.87	-7.1-2.4

30°/End-feel	73 ± 12	74 ± 10	0.70	0.36-0.87	-6.0-2.5

Calculations were made at three different flexion angles (90°, 60° and 30°) with four different applied torques (3, 6 and 9 Nm as well as the examiner's apprehension of end-feel) of the total internal-external knee joint rotation

The highest ICC_2.1 _was 0.87 at 90°, and 0.61-0.70 at 30° at 6 Nm applied torque, and the lowest 0.49 also at 90 but with at 3 Nm applied torque. The 95% CI between examiners was-7.1-3.8, except at 30° with 3 Nm (CI-7.9- -2.1).

## Discussion

As a result of the ICC calculations according to the recommendations of Fleiss [19], the Rottometer was judged to be a good (ICC 0.4-0.75) to excellent (ICC above 0.75) device concerning reliability for measuring knee rotation with 6 and 9 Nm applied torques and the examiner's apprehension of end-feel at three different flexion angles (90°, 60° and 30°). A torque of 3 Nm showed lower reliability in knee rotation measurements using the Rottometer as an assessment tool.

It seems as if the most valid [13] and reliable measurements of the knee rotation made by the Rottometer were registered at measurements of the total rotation at 9 Nm applied torque.

As measured with the Rottometer in present study, the range of total knee rotations at 9 Nm applied torque were 70°-79°. In earlier clinical in vivo studies measuring knee rotation, the total range of rotation have varied between 18° and 90° [7-12]. It may be argued that comparing measurement of knee rotation between different studies are of limited value, since different measurement devices, different flexion angles and different applied torques have been used. Also the set-ups during the examinations vary between different devices. However, as long as there is no accepted golden standard, further investigations of knee rotation both in healthy reference populations and in patients with different knee disorders and injuries, with valid as well as reliable measurement devices are needed.

### Sources of error

There are a number of sources of variability inherent in rotation measurements of the knee in vivo. A possible explanation of poor agreements at 3 Nm could be that measurements made with this torque produced a rather small range of rotation, and thus small differences between recordings resulted in relatively large disagreement. It is also possible that 3 Nm torque is too small to reach the end-points of the mechanical restraints and thus motions with a poorly defined end-point are measured. One aspect that could have affected the measurements at 60° and especially at 30° was that when the knee angle was changed and adjusted the hip angle was changed too, and thereby probably allowed movements in the hip [12], which could have been registered as knee rotation by the Rottometer. Careful consideration was given to the design of the fixation clamps [13] with the purpose of minimizing shifting due to soft tissue motion and at the same time avoiding pain, which makes it hard for the subject to relax during the examination. As in the within-day analyses, the design of the inter-tester measurements might have constituted a risk of a change in soft tissue characteristics or pain occurring, since two repeated measurements were made on the same subject on the same day. However, pain was not reported by any of the subjects at any time during the examinations. Unless the ends of the goniometer are surgically fixed to the bone, the potential for soft tissue movements nonetheless exists. On the other hand, pain or discomfort during the examination may induce muscle tension, which makes it impossible to measure the whole range of rotation. When different subjects endure different kinds of tightness of fixation this may cause a source of variability, as did the magnitude of each individual's soft tissue volume and elasticity. Positioning-related faults are differences in orientation of the goniometer between trials if the various clamps of the instrument are not positioned on the bones identically each time the goniometer is attached, which may lead to variations in starting position. This variation would cause problems in defining a starting "zero" resting position. During repeated non published pilot studies before the present investigation, we discovered a large individual variation of neutral knee rotation position between subjects, and the neutral position could also change due to different knee flexion angles within the subjects. The resting "zero" knee rotation position in Rottometer measurements was therefore defined as each individual subject's resting position of the knee at each flexion angle.

There may be a variation in the limits of passive motion from differences in elasticity and plasticity in the soft tissue depending on whether the measurements are made in the morning or afternoon, or whether the subjects have been taking part in any physical activity shortly before the examination. Maybe it is also possible that blood flow, temperature and stretching can influence the soft tissue characteristics, but such possible changes in rotation during the day were however not large enough to be registered with the device. However, during the intertester reliability test there was only a 10-min break between the two examinations, and that short break may have been a risk factor of soft tissue stretch effect.

The examinations were not blinded, and there is a risk that the examiners could have been influenced by earlier recordings or by measuring both knees on the same subjects. The risk of this kind of error is rather small due to the large number of recordings. The order of the different flexion angles (90°, 60° and 30°) and applied torques (3, 6, 9 Nm and end-feel) were not performed in random order due to technical and practical reasons, and that may have been a source of error. Different readings on the protractor between different examiners may also occur. It could be argued that the applied torque with the adjustable spanner might differ between examiners, if they were not experienced in using the equipment. In the present study the two examiners were however trained in using both the protractor and the spanner before the study, and did not consider using the equipment to be a problem. It is also possible that the flexion angle could cause variability between trials if the examiner is not careful to adjust to the exact angle each time.

However, despite all these possible errors, we believe that the Rottometer has the potential to be used in research and clinical practice, due to both its validity [13] and reliability. It may also be valuable to establish normal knee joint rotation reference values in a larger population of healthy individuals as well as to study possible age or gender differences.

## Conclusion

The Rottometer was judged to be a reliable measurement instrument concerning knee rotation at three different flexion angles (90°, 60° and 30°) at 6 and 9 Nm applied torques and as evaluated by the examiner's apprehension of end-feel. The most reliable measurements of the knee rotation made by the Rottometer were registered in measurements of the total rotation at 9 Nm applied torque. Measurements with 3 Nm were considered less reliable.

## Competing interests

The authors declare that they have no competing interests.

## Authors' contributions

P A conceived the manuscript, carried out the examination of the subjects, participated in the statistical analysis and participated in the design of the study, C E participated in the design and supervised the study, P-E I participated and supervised the statistical analysis, T F participated in the design and supervised the study. All authors read and approved the final manuscript.

## Pre-publication history

The pre-publication history for this paper can be accessed here:

http://www.biomedcentral.com/1471-2474/12/291/prepub
